# The effect of Proprioceptive Neuromuscular Facilitation (PNF) therapy on functional recovery in patients with knee joint injury: a systematic review and meta-analysis

**DOI:** 10.1186/s12891-025-09407-z

**Published:** 2025-12-17

**Authors:** Wenhua Zhang, Dianbo Zhang, Jun Liu

**Affiliations:** 1 College of Physical Education and Health, Guangxi Normal University, Guilin, Guangxi China; 2 School of Sport Communication and Information Technology, Shandong Sport University, Jinan, Shandong China; 3 Sports Science Research Institute, Shandong Sport University, No. 10600 Century Avenue, Licheng District, Jinan City, Shandong Province 250102 China

**Keywords:** Proprioceptive neuromuscular facilitation, Knee joint injury, Functional recovery, Rehabilitation exercise, Randomized controlled trials, Meta-analysis

## Abstract

**Objective:**

This study employed a meta-analytic approach to systematically evaluate the effects of proprioceptive neuromuscular facilitation (PNF) therapy on functional recovery in patients with knee joint injuries.

**Methods:**

Randomized controlled trials investigating the effects of PNF on functional recovery in patients with knee joint injuries were retrieved from the China National Knowledge Infrastructure, PubMed, Embase, Cochrane Library, and Web of Science databases. The methodological quality of the included studies was assessed using the Cochrane Risk of Bias Assessment Tool. Meta-analysis was performed using RStudio, Review Manager 5.4 and Stata 17 software. Pooled effect sizes were calculated as weighted mean differences (WMDs) or standardized mean differences (SMDs) with corresponding 95% confidence intervals (CIs) and 95% prediction intervals (PIs). Heterogeneity was evaluated using the *I²* statistic. A fixed-effects model was applied when *I²* ≤ 50%, whereas a random-effects model was used when substantial heterogeneity was present (*I²* > 50%).

**Results:**

A total of 13 studies involving 762 participants were included. PNF demonstrated significant benefits in patients with knee injuries, including improvements in knee flexion range of motion (WMD = 11.09, 95%CI: 4.60–17.57, 95%PI: -10.73–32.90), extension range of motion (WMD = 5.42, 95% CI: 1.29–9.56, 95%PI: -40.77–51.61), pain reduction (SMD = -0.93, 95% CI: -1.68 – -0.19, 95%PI: -4.01–2.14)), Lysholm Knee Scoring Scale scores (WMD = 13.96, 95% CI: 6.44–21.49, 95%PI: -17.63–45.55), Berg Balance Scale scores (WMD = 2.83, 95% CI: 0.49–5.17, 95%PI: -21.54–27.20), and Activities of Daily Living scores (SMD = 0.51, 95% CI: 0.12–0.91, 95%PI: -0.79–1.81). Subgroup analyses suggested that combined PNF interventions of 8–12 weeks, performed six times per week for 10–30 min per session, were associated with the greatest improvements in knee joint range of motion during traumatic or postoperative recovery. Similarly, the most pronounced reductions in pain were observed with combined PNF programs of 8–12 weeks, delivered 5–7 times per week for 10–30 min per session, were more effective in alleviating pain in this population.

**Conclusions:**

PNF can significantly improve the active flexion range, extension range, pain, knee joint function, balance ability, and activities of daily living in patients with knee disorders. More high-quality studies with larger samples are still needed for further research.

**Supplementary Information:**

The online version contains supplementary material available at 10.1186/s12891-025-09407-z.

## Introduction

With the rapid expansion of public fitness participation, sports-related knee injuries have become increasingly prevalent [1]. These injuries often result from high-impact activities performed without proper guidance, combined with excessive training intensity [2, 3]. In the context of the Healthy China initiative, the integration of sports and medicine has been emphasized to promote scientific fitness and improve injury prevention and rehabilitation [4].

The knee joint, the largest and most complex in the human body, connects the femur, tibia, and patella, primarily enables flexion and extension with limited rotation, relies on ligaments, menisci, and surrounding musculature for stability, and is particularly prone to injury and degenerative changes [5]. Compared with other musculoskeletal injuries such as muscle strains or tendinopathies, knee injuries typically involve multiple anatomical structures simultaneously (e.g., ligaments, menisci, and articular cartilage), leading to a combination of mechanical instability, altered load distribution, and proprioceptive deficits [6–8]. This multi-structure involvement makes knee injuries more complex in both diagnosis and rehabilitation, as they not only cause localized symptoms but also disrupt lower limb kinetic chains, influencing hip and ankle biomechanics [8, 9]. It is noteworthy that among various knee joint disorders, knee osteoarthritis (KOA) is a chronic degenerative joint disease characterized by articular cartilage degeneration, osteophyte formation, and synovial inflammation, primarily manifesting as pain, stiffness, and limited mobility [10]. As patients with KOA often exhibit muscle imbalances and impaired proprioception [7]. Rehabilitation interventions should comprehensively target both muscle function and neural control.

Against this background, proprioceptive neuromuscular facilitation (PNF), as a rehabilitation and exercise training approach grounded in neurophysiological principles, is commonly employed in the recovery of knee joint injuries [11]. By utilizing specific spiral-diagonal movement patterns, resistance application, and techniques of muscle contraction and relaxation, PNF activates the proprioceptive system to enhance neuromuscular control, strength, flexibility, and coordination [11–13]. Widely applied in both rehabilitation medicine and athletic performance optimization, PNF emphasizes integrative training of multiple joints and muscle groups to improve functional movement capacity [14]. PNF, by integrating isometric contractions with passive stretching, can effectively enhance muscle flexibility, joint range of motion, and alleviate pain in patients with knee joint injuries. Its underlying mechanism is grounded in neurophysiological principles, such as activation of the Golgi tendon organs (GTOs) to induce autogenic inhibition, thereby reducing muscle resistance and augmenting the stretching effect [13]. Evidence indicates that PNF significantly decreases pain and improves hamstring flexibility in patients with KOA, demonstrating superior outcomes compared to static stretching [15, 16]. Moreover, PNF may help balance medial and lateral knee joint pressures by modulating stress distribution, thereby potentially slowing the progression of degenerative changes [17].

PNF alone has been shown to produce significantly greater improvements in flexibility (e.g., hamstring range of motion) compared to static stretching, suggesting its superiority in inducing neuromuscular relaxation and lengthening [18]. In patients with low back pain, meta-analytic evidence demonstrates that PNF training yields large effect sizes in reducing both pain and functional disability compared to other physical therapy interventions [19]. In stroke rehabilitation, systematic review data indicate that PNF produces more pronounced gains in gait parameters than routine physiotherapy, likely by leveraging enhanced proprioceptive input and motor control [20].

PNF has shown significant short-term and long-term improvements in joint range of motion and muscle flexibility in older adults, suggesting potential applications in mobility maintenance and injury prevention [21]. For athletes with chronic ankle instability, PNF has been found to enhance balance, strength, and reduce pain, indicating that it may play a role in injury recovery and performance enhancement [22]. 

In recent years, although some reviews have systematically summarized rehabilitation interventions for patients with knee joint injuries [23, 24], PNF has typically been considered only as one of multiple interventions, and its specific effects on outcomes such as joint range of motion, balance, and activities of daily living have not been independently investigated. Although Zhu et al. compared various stretching methods, their discussion of PNF was relatively broad and did not delve into its specific protocols [24]; Zhao et al. primarily focused on the overall effects of aquatic exercise, where PNF was only one component of the intervention, and its individual contribution was not independently assessed [23]. Therefore, this study holds greater advantages in terms of the depth of specialized PNF research, methodological rigor, and the specificity of clinical guidance. This study systematically searched both domestic and international databases for randomized controlled trials (RCTs) evaluating PNF in patients with knee joint injuries. A systematic review and meta-analysis were conducted to determine whether PNF provides additional benefits compared with conventional rehabilitation, thereby offering evidence to support the application and promotion of PNF in the rehabilitation of knee joint injury patients.

## Methods

### Protocol and registration

This comprehensive systematic review and meta-analysis was conducted in accordance with the guidelines outlined in the Preferred Reporting Items for Systematic Reviews and Meta-Analyses (PRISMA) 2020 statement [25](Supplementary Table S1). The study has been prospectively registered with PROSPERO under the registration number CRD420251145936.

### Search strategy

The literature search was conducted by two investigators (W.Z. and J.L.) starting in September 2025. The databases searched included China National Knowledge Infrastructure (CNKI), PubMed, Embase, Cochrane Library and Web of Science. RCTs investigating the effects of PNF on functional recovery in patients with knee injuries were retrieved from each database. The search terms included “proprioceptive neuromuscular facilitation,” “neuromuscular,” “proprioception,” “neuromuscular facilitation,” “proprioceptive facilitation,” “rhythmic stabilization,” “reciprocal inhibition,” “spiral diagonal,” “antagonist contraction,” “repeated contraction,” “knee injury,” “knee sports injury,” “knee osteoarthritis,” and “randomized controlled trial.” To identify more potential studies, we manually searched gray literature, reference lists of identified studies, and relevant registration website (ClinicalTrials.gov) and consulted experts in this field. however, due to the lack of standardized peer-review processes and limited accessibility of detailed data, we decided to exclude gray literature from our analysis. The full search strategies for all databases are shown in Supplementary Table S2.

### Inclusion and exclusion criteria for the studies

Inclusion Criteria: (1) Study Type: RCTs published in various databases from their inception to September 2025 investigating the effects of PNF on functional recovery in patients with knee injuries. (2) Subjects: Patients with clinically confirmed knee joint injuries, diagnosed based on at least one of the following objective criteria and accompanied by corresponding clinical symptoms and signs (such as pain, swelling, limited mobility, etc.): (i) Imaging evidence, such as X-ray showing joint space narrowing, osteophyte formation, etc., or magnetic resonance imaging indicating ligament, meniscus, or cartilage injury, or abnormal ultrasound findings [26]; (ii) Confirmation by arthroscopy; (iii) Meeting the American College of Rheumatology diagnostic criteria for knee osteoarthritis, or having a typical history and signs of sports injuries such as anterior cruciate ligament rupture or meniscal tear, confirmed by a specialist physician [27]. All patients were clinically stable, able to cooperate with rehabilitation therapy, had no cognitive or language barriers, could communicate effectively with the therapist and actively participate in PNF treatment, and the patients or their family members had signed informed consent. Knee joint injuries are specifically categorized into acute traumatic injuries (e.g., anterior cruciate ligament rupture, meniscal tear, etc.), chronic degenerative diseases (e.g., knee osteoarthritis), and postoperative recovery cases (e.g., following total knee arthroplasty). (3) Interventions: The control group received conventional rehabilitation, routine care, pharmacological treatments, or other interventions. The experimental group received PNF-based training in addition to the control interventions or underwent PNF training alone. PNF training could include various modalities, such as D1 and D2 patterns, resistance training, or aquatic exercises. (4) Outcome Measures: Primary and secondary outcomes included Knee Active Flexion Range (FR) and Active Extension Range (ER); Visual Analogue Scale (VAS); Berg Balance Scale (BBS); Knee Injury and Osteoarthritis Outcome Score (KOOS); Lysholm Knee Scoring Scale (LKSS); Hospital for Special Surgery (HSS) Knee Score; Western Ontario and McMaster Universities Osteoarthritis Index (WOMAC); Numeric Pain Rating Scale (NPRS); Functional Independence Measurement (FIM); and Activities of Daily Living (ADL). (5) Language: The publication language of the studies was restricted to Chinese and English academic journals.

Exclusion Criteria: Studies were excluded if they met any of the following conditions: (1) duplicate publications; (2) concomitant major organ disease, including but not limited to cardiac or pulmonary disorders; (3) instances of secondary injury to the knee joint; (4) any contraindications to hydrotherapy, such as communicable diseases or the presence of open wounds; (5) receipt of surgical or other invasive treatments over the course of the investigation; or (6) diagnosed psychological or phobic conditions, for example aquaphobia, that could interfere with the intervention.

### Literature screening and data extraction

The retrieved literature was imported into EndNote 20 (Clarivate Analytics, USA), and duplicates were removed. Subsequently, two researchers (W.Z. and D.Z.) independently screened the studies and extracted relevant information based on predefined inclusion and exclusion criteria. Any discrepancies were resolved through discussion with a third researcher. The characteristics of the included studies encompassed the authors, publication year, country, type of knee injury, sample size, participant age and interventions in the experimental and control Groups (Table 1). Intervention characteristics included PNF intervention, frequency of PNF therapy, duration of the PNF intervention program, duration of each PNF session, control group interventions, and outcome measures (Table 2).

**Table 1:** Research characteristics

Author (Year)	Country and area	Types of Knee Joint Injuries	Sample size (*n*)		Age (year)		Research Group	
			EG	CG	EG	CG	EG	CG
Wu (2008) [31]	China	Distal femoral fracture, Tibial plateau fracture, Meniscal injury	40	40	42.07 ± 13.95	41.27 ± 12.85	PNF+①	①
Weng (2009) [32]	China, Taiwan	KOA	62	62	64.0 ± 7.5	64.0 ± 7.5	PNF+②	②
Wang (2009) [33]	China	Fractures (tibia, tibial plateau, patella), soft tissue injuries (meniscus, ligaments), and fractures complicated by concomitant soft tissue injuries	40	40	32.6 ± 3.5		Aquatic PNF+③	③
Hao (2010) [34]	China	Distal femoral fractures, intercondylar femoral fractures, tibial plateau fractures, and patellar fractures	37	32	35 ± 2.9	33 ± 3.1	PNF+③+④	③+④
Wang (2013) [35]	China	Reconstruction Following Isolated Ligament injury and surgery after fracture	15	15	41.15 ± 19.68	38.94 ± 20.01	PNF+①	①
Yin (2018) [36]	China	KOA	25	25	68 ± 7	69 ± 7	PNF	⑨
Jaczewska-Bogacka (2018) [38]	Poland	KOA	28	28	68.7 ± 8.8	68.1 ± 6.9	PNF	⑤
Li (2018) [37]	China	KOA	30	30	63.5 ± 8.8	64.3 ± 9.2	PNF+⑥	⑥
Tao (2018) [39]	China	KOA	29	30	63.80 ± 8.15	65.00 ± 7.39	PNF	⑨
Song (2020) [40]	China	KOA	13	16	68.5 ± 4.3	67.4 ± 3.4	PNF	⑦
Shen (2022) [41]	China	KOA	14	13	65.3 ± 4.6	66.6 ± 7.0	PNF	⑦
Zhang (2023) [42]	China	KOA	20	20	56.55 ± 4.01	57.85 ± 4.10	PNF+⑧	⑧
Markopoulos (2025) [43]	Greece	KOA	28	30	67.50 ± 2.83	67.37 ± 3.04	PNF	⑤

*EG* Experimental Group, *CG* Control Group, *KOA* Knee osteoarthritis, *PNF* Proprioceptive Neuromuscular Facilitation

①, therapeutic joint mobilization

②, isokinetic muscle strength training

③, systematic comprehensive rehabilitation

④, Chinese Medicine smoke therapy

⑤, conventional home-based exercise program

⑥, electrocaloric and magnetocaloric effects

⑦, Health education

⑧, neuromuscular exercise

⑨, routine rehabilitation training

**Table 2:** Characterization of research interventions

Athor	PNF intervention	Frequency of intervention	Intervention cycle	Duration of one intervention	Outcome measures
Wu [31]	HR and CR, each technique is repeated 10 times, with each resisted hold maintained for 10 s.	NR	2 mouths	NR	FR, ER
Weng [32]	HR and CR, each stretching step was performed for 15 s, and the entire sequence was repeated 10 times per session	3 times/week	8 weeks	10 min	FR, VAS
Wang [33]	HR and CR	7 times/week	8 weeks	20 min	VAS, FIM
Hao [34]	HR, CR, DR, and RS	6 times/week	2 weeks	20 min	ER, FR, VAS
Wang [35]	D2-F pattern of lower limb flexion and D2-E pattern of lower limb extension	7 times/week	12 weeks	20 min	VAS, LKSS
Yin [36]	D2-F pattern of lower limb flexion and D2-E pattern of lower limb extension, 10 repetitions per set, 2 sets; SR, each resistance change was maintained for 5 s, 10 changes constituted one set, for a total of 3 sets	5 times/week	4 weeks	30 min	BBS, HSS
Jaczewska-Bogacka [38]	HR, CR, DR, RI, RS, SR, and COI, 10 times	3 times/week	3 weeks	75 min	VAS
Li [37]	HR, CR, DR, RS	5 times/week	2 weeks	30 min	VAS, LKSS
Tao [39]	D1-F pattern of lower limb flexion, D1-E pattern of lower limb extension, D2-F pattern of lower limb flexion and D2-E pattern of lower limb extension	5 times/week	4 weeks	40 min	VAS, LKSS
Song [40]	D1-F pattern of lower limb flexion, D1-E pattern of lower limb extension, D2-F pattern of lower limb flexion and D2-E pattern of lower limb extension, each pattern was performed for 5–8 repetitions per set, with a total of 3 sets per session, HR, CR, ROA, and repeated stretch	3 times/week	12weeks	45 min	FR, WOMAC
Shen [41]	D1-F pattern of lower limb flexion, D1-E pattern of lower limb extension, D2-F pattern of lower limb flexion and D2-E pattern of lower limb extension, HR, CR, ROA	3 times/week	6 weeks	45 min	FR, WOMAC
Zhang [42]	D1-F pattern of lower limb flexion, D1-E pattern of lower limb extension, D2-F pattern of lower limb flexion and D2-E pattern of lower limb extension, HR, CR, RI, DR, SR, RS, COI	2 times/week	6 weeks	60 min	FR, VAS, KOOS
Markopoulos [43]	Spiral and diagonal pattern, HR, RI, SR, DR, RS, COI	3 times/week	6 weeks	45 min	FR, NPRS, KOOS, BBS

*EG* Experimental group, *CG* Control group, *PNF* Proprioceptive neuromuscular facilitation, *NEMEX* Neuromuscular exercise, *VAS* Visual analog scale, *FR* Flexion range, *ER* Extension range, *BBS* Berg balance scale, *KOOS* Knee injury and osteoarthritis outcome score, *LKSS* Lysholm Knee Score Scale, *HSS* Hospital for Special Surgery, *WOMAC* Western Ontario and McMaster Universities Osteoarthritis Index, *NPRS* Numeric Pain Rating Scale, *FIM* Functional Independence Measurement, *HR* Hold-Relax, *CR* Contract-Relax, *DR* Dynamic Reversal, *RS* Rhythmic Stabilization, *D2-F* Diagonal 2-Fexion, *D2-E* Diagonal 2-Extension, *RI* Stabilizing Initiation, *SR* Stabilizing Reversals, *COI* Combination of Isotonic, *ROA* Reversal of Antagonists, *NR* Not Report

Outcome data assessed at the immediate conclusion of the intervention were prioritized for extraction. For studies reporting multiple follow-up time points, the latest available data from the end of the primary intervention period were selected to evaluate PNF’s immediate and short-term effects. All continuous data were extracted as post-intervention means and standard deviations (SD). In multi-arm trials: data from independent PNF intervention groups were extracted separately, while combined PNF therapy groups were treated as a single intervention unit and compared against the conventional rehabilitation control group to maintain intergroup independence.

### Risk of bias assessment

For RCTs and clinical controlled trials, the risk of bias was assessed using the built-in tool in Review Manager 5.4 [28]. Two independent reviewers evaluated the risk of bias of the included studies with the Cochrane Risk of Bias Assessment Tool. The domains assessed included random sequence generation, allocation concealment, blinding of participants and investigators, blinding of outcome assessment, completeness of outcome data, selective reporting, and other potential sources of bias. Each domain was rated as having a high, low, or unclear risk of bias. Any discrepancies between reviewers were resolved through discussion.

### Data analysis

Forest plots with 95% prediction intervals (95% PIs) were generated using RStudio. Subgroup analysis and risk of bias assessment were performed with Review Manager 5.4. Sensitivity analysis, funnel plots, and Egger’s test were conducted using Stata 17. For continuous outcomes measured in identical units, effect sizes were calculated using weighted mean differences (WMDs) with corresponding 95% confidence intervals (95% CIs). When different measurement tools were employed, standardized mean differences (SMDs) was used to pool effect sizes. Statistical inference was based on tests of heterogeneity and pooled effect estimates. Heterogeneity was assessed using the *I²* statistic. A value of *P* > 0.10 was considered indicative of negligible heterogeneity, whereas *P* ≤ 0.10 suggested the presence of heterogeneity among the included studies. The thresholds for *I²* were interpreted as follows: 0–25% indicated negligible heterogeneity, 25–50% mild heterogeneity, 50–75% moderate heterogeneity, and > 75% substantial heterogeneity [29]. Random-effects models were applied when moderate to substantial heterogeneity was detected, while fixed-effects models were used when heterogeneity was mild or negligible.

### Subgroup analysis

Subgroup analyses of FR and pain scores were conducted according to injury type, intervention modality, intervention duration, intervention frequency, and single-session intervention time. Pain scores were further stratified by different assessment scales. Due to the limited number of studies, no subgroup analyses were performed for other outcome measures.

### Sensitivity analysis

We conducted a sensitivity analysis by sequentially excluding individual studies to verify the robustness of the results.

### Publication bias

To assess publication bias, when a meta-analysis included ≥ 10 studies reporting the same outcome measure, we evaluated potential bias using funnel plots and Egger’s test.

### Meta-regression analysis

To further explore the sources of heterogeneity (*I²* > 50%) among the included studies, a meta-regression analysis was conducted on potential contributing factors. Since only the outcome of pain scores was assessed in ≥ 10 studies, it met the methodological requirement for such analysis. Four variables—type of injury, type of intervention, duration of intervention, and intervention frequency—were included as covariates in a univariate meta-regression analysis.

### Certainty of evidence

We applied the Grading of Recommendations Assessment, Development, and Evaluation (GRADE) system to assess the certainty of evidence. Each outcome was evaluated across six domains: study design, risk of bias, inconsistency, indirectness, imprecision, and other considerations. Subsequently, the certainty of evidence was classified as “high,” “moderate,” “low,” or “very low” [30]. The GRADE pro GDT online tool was used to present the summary of findings.

## Results

### Results of literature screening

**Fig. 1:**
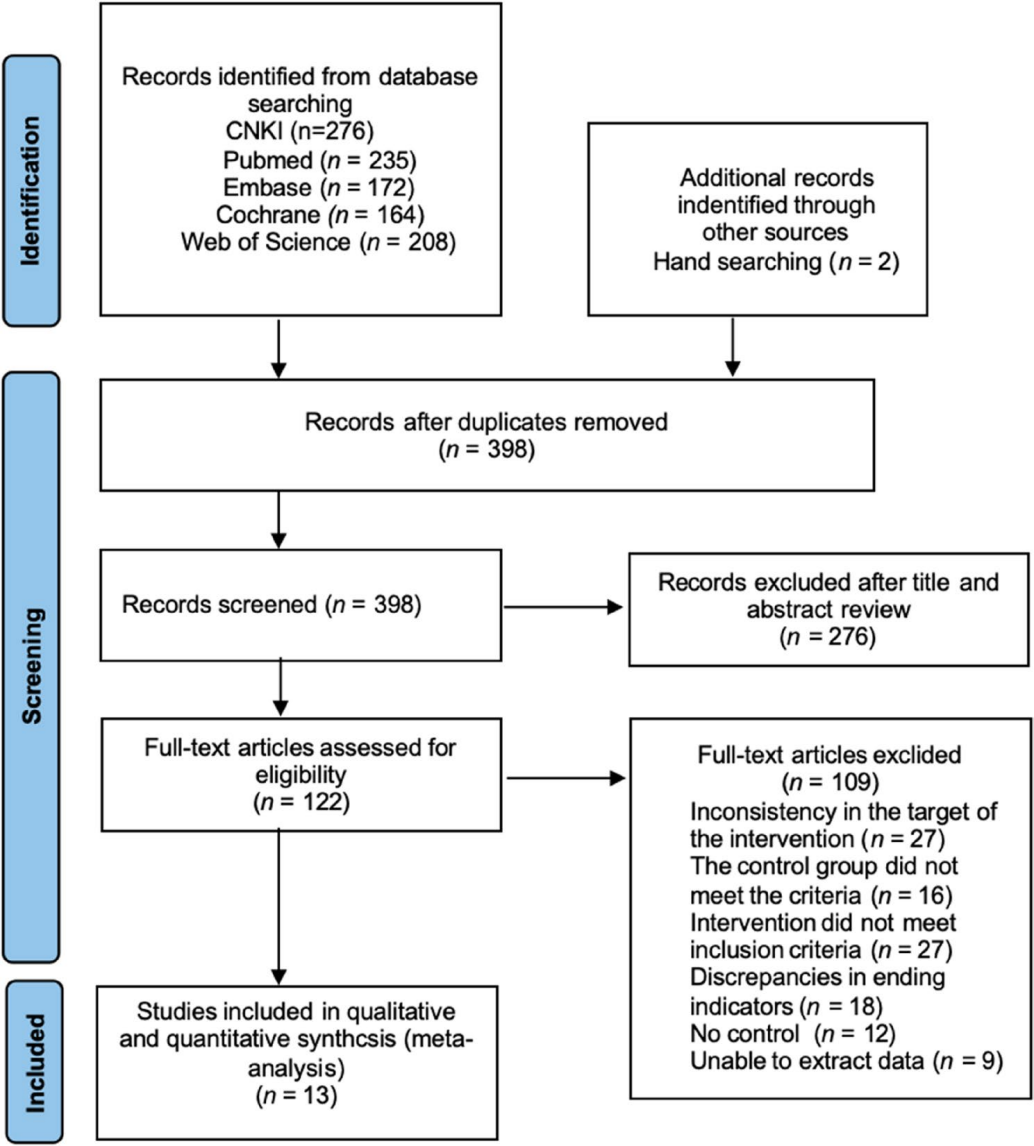
PRISMA study flow diagram

A total of 1,057 relevant articles were initially identified, including 276 from the CNKI database, 235 from PubMed, 172 from Embase, 164 from the Cochrane Library, 208 from Web of Science, and 2 from other sources. After removing duplicates using EndNote 20, 398 articles remained. Screening of titles and abstracts led to the exclusion of 276 articles, leaving 122 for full-text review to assess eligibility. Among these, 27 studies involved ineligible intervention subjects, 16 had control groups that did not meet the criteria, 27 employed interventions inconsistent with the inclusion standards, 18 reported outcomes that did not meet the requirements, 12 lacked a control group, and 9 did not provide extractable data. Ultimately, 13 studies were included in the meta-analysis [31–43] (Fig. 1).

### Characteristics of the included studies

This study included a total of 13 articles published between 2008 and 2025, involving 762 participants (381 in the experimental groups and 381 in the control groups). Among these publications, 10 originated from mainland China [31, 33–37, 39–42], one from Taiwan [32], one from Poland [38], and one from Greece [43]. Most studies (9/13) recruited patients with KOA [32, 36–43]. With respect to the intervention strategies, seven studies employed PNF combined with other interventions (Joint mobilization, isokinetic muscle strength training, neuromuscular exercise training, traditional Chinese medicine therapies, electrothermal and magnetothermal therapy) [31–35, 37, 42], while six studies applied PNF training alone [36, 38–41, 43]. The intervention duration ranged from 2 to 12 weeks, with a frequency of 2 to 7 sessions per week and a session length of 10 to 70 min (Tables 1 and 2).

### Risk of bias

**Fig. 2:**
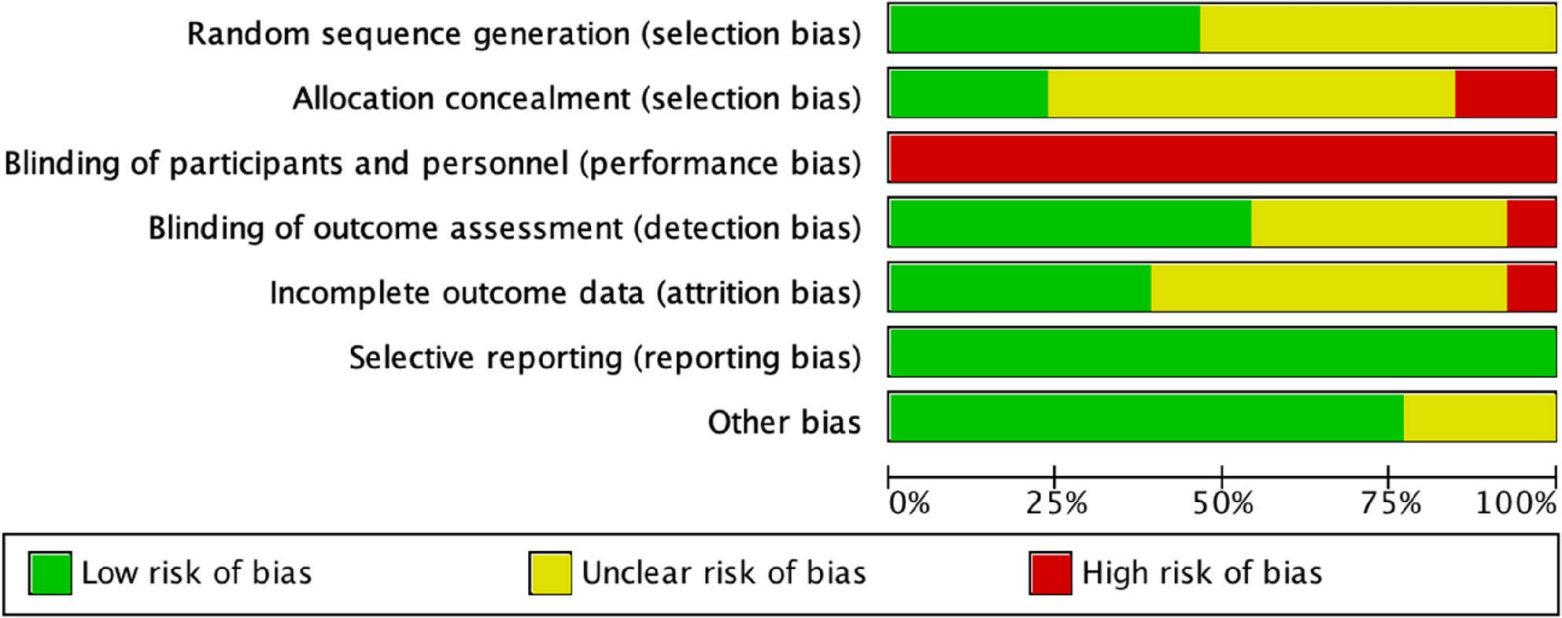
Risk of bias of the included studies

**Fig. 3:**
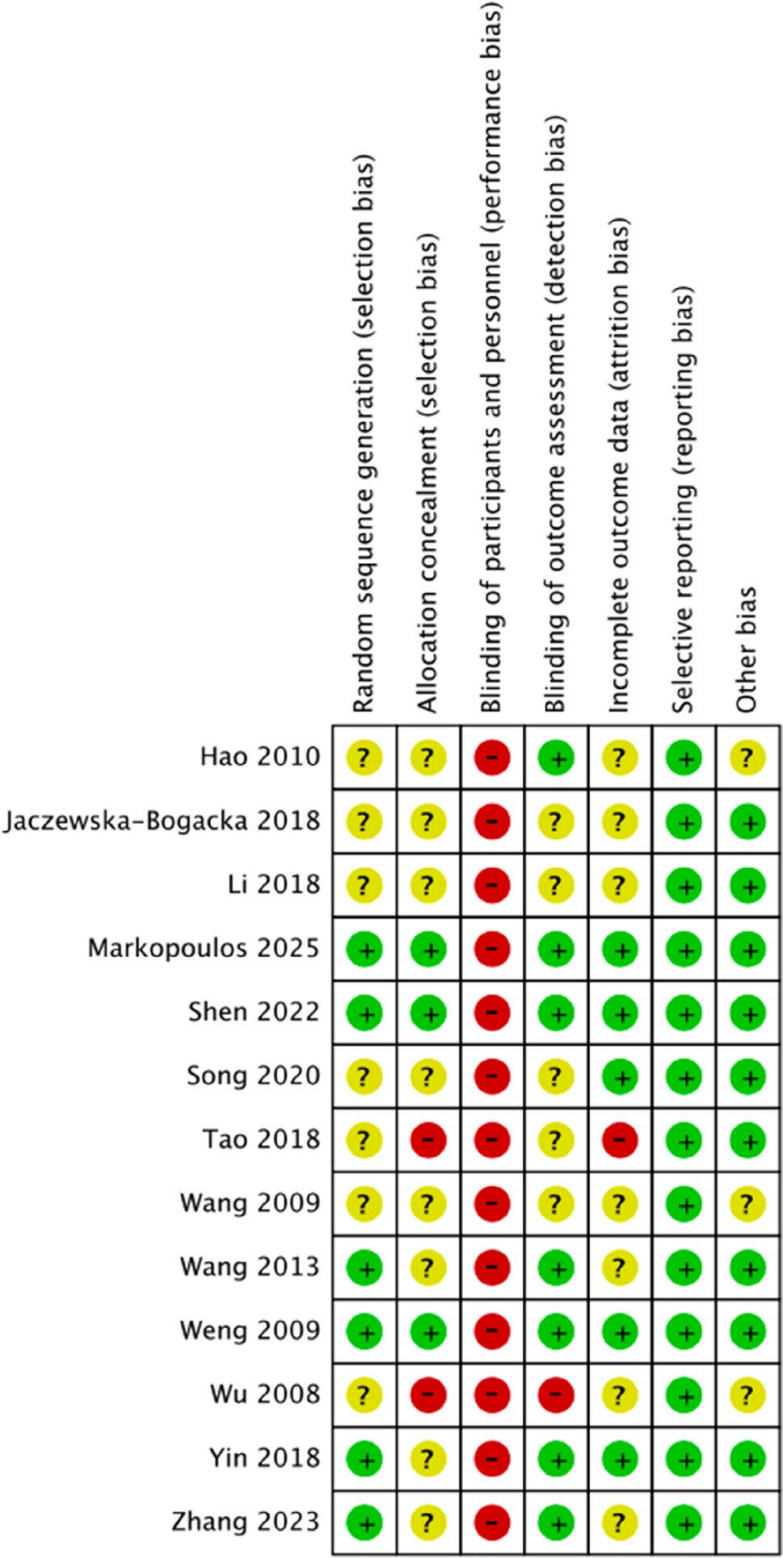
Risk of bias summary of the included studies

Six studies [32, 35, 36, 41–43] reported methods for generating random sequences, including lottery drawing, random number tables, computer-generated sequences, and block randomization. Two studies [32, 43] provided details on allocation concealment—one using consecutively numbered, opaque, sealed envelopes, and the other conducted by an independent researcher to ensure concealment. Five studies [32, 39–41, 43]reported the number of dropouts and losses to follow-up. Two studies [32, 43] conducted intention-to-treat (ITT) analyses, while the remaining studies did not explicitly state whether ITT or per-protocol (PP) analyses were applied. One study [43] reported a trial registration number. Overall, due to the inherent difficulty of blinding in PNF interventions, all studies were considered at high risk of bias. The results of the risk-of-bias assessment are presented in Figs. 2 and 3.

### Meta-analysis results

#### Effects of PNF on knee joint range of motion

##### Effect of PNF on FR

**Fig. 4:**
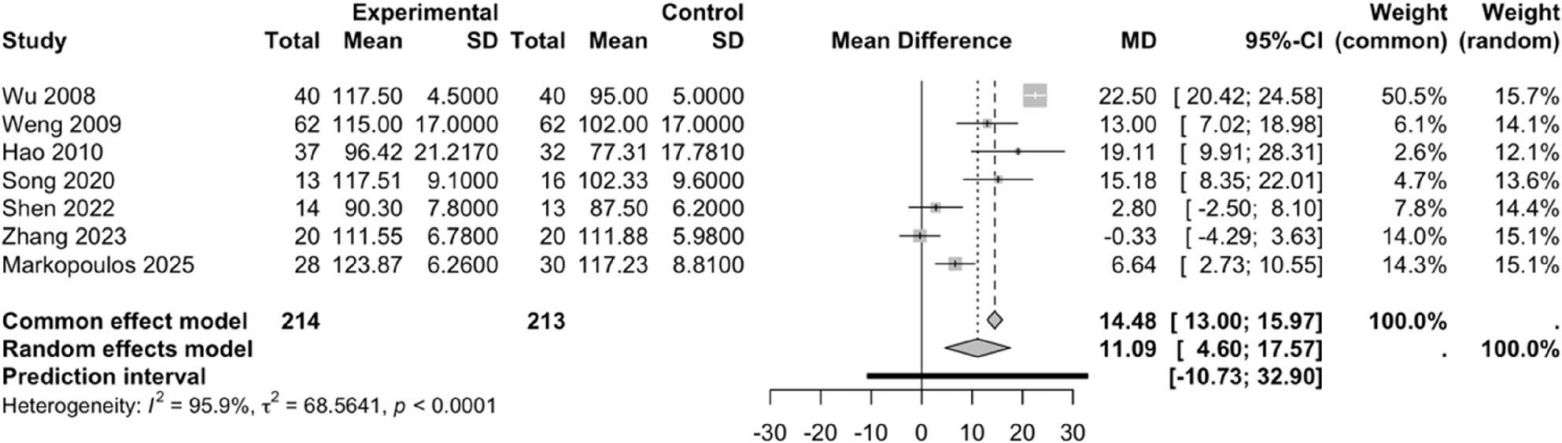
Forest plot of the effect of PNF on FR

Seven studies [31, 32, 34, 40–43] included in this analysis investigated the effect of PNF on FR. Comparison between the experimental and control groups revealed substantial heterogeneity among the included studies (*I²* = 95.9%, *P* < 0.0001); therefore, a random-effects model was applied. PNF significantly improved FR in patients with knee injuries compared with the control group (WMD = 11.09, 95% CI: 4.60–17.57, 95% PI: -10.73–32.90) (Fig. 4).

Subgroup analyses were conducted based on potential sources of heterogeneity (Table 3). The included studies were categorized by type of injury into two groups: acute traumatic or postoperative injuries, and chronic degenerative conditions. By intervention type, three studies [40, 41, 43] applied PNF alone, while four studies [31, 32, 34, 42] combined PNF in combination with other interventions; accordingly, they were divided into the subgroups of PNF alone and PNF in combination with other interventions. Regarding the intervention duration, studies were grouped into 2–6 weeks and 8–12 weeks. For intervention frequency, two subgroups were defined: 2–3 sessions/week and 6 sessions/week (excluding one study [31] that did not specify frequency). Finally, in terms of session length, interventions were divided into 10–30 min and 40–60 min per session (excluding one study [31] that did not specify session duration).

**Table 3:** Subgroup analysis of the effects of PNF on knee joint FR in patients with knee injuries

Research characteristics		Number of experiments	WMD [95%CI]	The *P*-value of WMD	I^2^(%)	The *P*-value of I2
Injury types	Recovery period for trauma or postoperative injury	2	22.33 [20.30, 24.37]	< 0.00001	0	0.48
	Chronic Degenerative Disorders	5	7.07 [1.67, 12.48]	0.01	83	0.0001
Intervention modality	PNF alone	3	7.80 [1.75, 13.85]	0.01	75	0.02
	PNF in combination with other interventions	4	13.48 [0.69, 26.26]	0.04	97	< 0.00001
Intervention cycle	2–6 weeks	4	5.99 [-0.05, 12.02]	0.05	82	0.0008
	8–12 weeks	3	17.40 [10.59, 24.21]	< 0.00001	83	0.003
Frequency of intervention	2–3 times/week	5	7.07 [1.67, 12.48]	0.01	83	0.0001
	6 weeks/weeks	1	19.11 [9.91, 28.31]	< 0.0001		
Duration of one intervention	10–30 min	2	15.01 [9.38, 20.64]	< 0.00001	16	0.28
	40–60 min	4	5.65 [-0.02, 11.32]	0.05	82	0.0008

*WMD* Weighted mean difference, *CI* Confidence interval, *PNF* Proprioceptive Neuromuscular Facilitation

The subgroup analysis revealed that in patients during the recovery phase following trauma or postoperative injury, interventions lasting 10–30 min per session showed substantially reduced heterogeneity compared with the overall pooled effect (*I²* = 96%), while yielding statistically significant results. Specifically, the heterogeneity within these subgroups was 0% and 16%, respectively. These findings suggest that the above subgroups may account for the source of the high overall heterogeneity.

Subgroup analysis (Table 3) indicated that the largest effect sizes for improving FR were observed under the following intervention parameters: a duration of 8–12 weeks (WMD = 17.40, 95% CI: 10.59–24.21, *P* < 0.00001), a frequency of 6 sessions per week (WMD = 19.11, 95% CI: 9.91–28.31, *P* < 0.00001), and a session length of 10–30 min (WMD = 15.01, 95% CI: 9.38–20.64, *P* < 0.00001). In addition, combined PNF interventions overall demonstrated a significant benefit (WMD = 13.48, 95% CI: 0.69–26.26, *P* = 0.04), particularly for patients during the post-trauma or postoperative recovery phase (WMD = 22.33, 95% CI: 20.30–24.37, *P* < 0.00001). It should be noted that these patterns are exploratory and derived from a limited number of studies within each subgroup.

##### Effect of PNF on ER

**Fig. 5:**
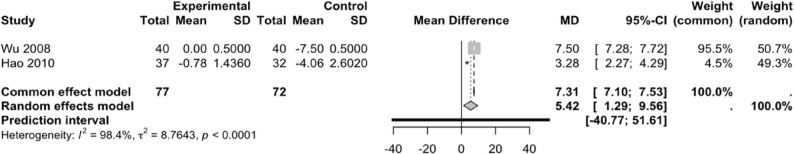
Forest plot of the effect of PNF on ER

Two included studies [31, 34] reported the effects of PNF on ER of the knee joint. A high degree of heterogeneity was observed across the study results (*I²* = 98.4%, *P* < 0.0001). Therefore, a random-effects model was applied for analysis. PNF significantly improved ER in patients with knee joint injuries compared with the control group (WMD = 5.42, 95% CI: 1.29–9.56, 95% PI: -40.77–51.61) (Fig. 5).

#### Effect of PNF on pain scores

**Fig. 6:**
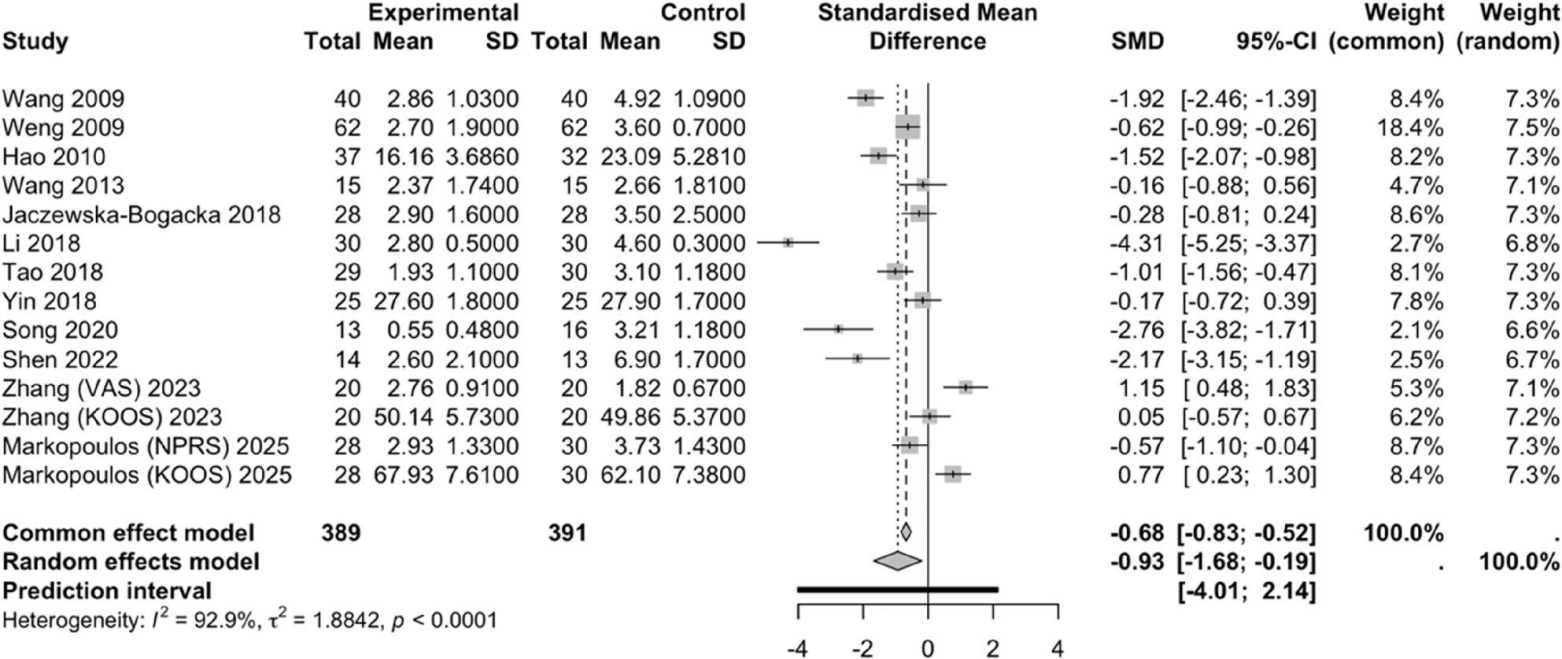
Forest plot illustrating the effect of PNF on pain scores

A total of 12 studies [32–43] included in this analysis investigated the effects of PNF on pain scores. Comparison between the intervention and control groups revealed substantial heterogeneity across studies (*I²* = 92.9%, *P* < 0.0001); therefore, a random-effects model was applied. Among the included studies, eight used the VAS [32–35, 37–39, 42], two used the WOMAC [40, 41], two used the KOOS [42, 43], one used the HSS score [36], and one used the NPRS [43]. SMD were calculated because all scales have lower scores indicating less pain. PNF significantly reduced pain scores in patients with knee injuries compared to controls (SMD = -0.93, 95% CI: -1.68 – -0.19, 95% PI: -4.01–2.14) (Fig. 6). Additionally, the use of different pain assessment scales may have contributed to the observed heterogeneity.

Subgroup analyses were performed to explore potential sources of heterogeneity (Table 4). Studies were stratified by injury type (traumatic or postoperative vs. chronic degenerative), intervention modality (PNF alone vs. PNF in combination with other interventions), intervention duration (2–6 weeks vs. 8–12 weeks), frequency (2–3 vs. 5–7 sessions/week), and single-session length (10–30 vs. 40–75 min). While heterogeneity decreased in some subgroups, high heterogeneity remained overall, suggesting that the primary sources of heterogeneity could not be fully accounted for through these subgroup analyses.

**Table 4:** Subgroup analysis of the effects of PNF on pain scores in patients with knee injuries

Research characteristics		Number of experiments	SMD [95%CI]	The *P*-value of SMD	I^2^(%)	The *P*-value of I^2^
Scale Type	VAS	8	-1.34 [-2.01, -0.67]	< 0.0001	91	< 0.00001
	KOOS	2	0.43 [-0.28, 1.13]	0.23	66	0.09
	WOMAC	2	-2.45 [-3.17, -1.73]	< 0.00001	0	0.42
	HSS	1	-0.17 [-0.72, 0.39]	0.55	—	—
	NPRS	1	-0.57 [-1.10, -0.04]	0.03	—	—
Injury types	Recovery period for trauma or postoperative injury	3	-1.23 [-2.18, -0.28]	0.01	87	0.0005
	Chronic Degenerative Disorders	9	-1.05 [-1.68, -0.41]	0.001	92	< 0.00001
Intervention modality	PNF alone	6	-0.80 [-1.51, -0.08]	0.03	89	< 0.00001
	PNF in combination with other interventions	6	-1.35 [-2.17, -0.54]	0.001	93	< 0.00001
Intervention cycle	2–6 weeks	8	-1.00 [-1.70, -0.30]	0.005	92	< 0.00001
	8–12 weeks	4	-1.31 [-2.28, -0.33]	0.008	90	< 0.00001
Frequency of intervention	2–3 times/week	6	-0.77 [-1.38, -0.15]	0.01	88	< 0.00001
	5–7 weeks/weeks	6	-1.48 [-2.42, -0.53]	0.002	93	< 0.00001
Duration of one intervention	10–30 min	6	-1.40 [-2.32, -0.49]	0.003	94	< 0.00001
	40–75 min	6	-0.83 [-1.50, -0.16]	0.02	89	< 0.00001

*SMD* Standardized mean difference, *CI* Confidence interval, *PNF* Proprioceptive Neuromuscular Facilitation, *VAS* visual analog scale, *KOOS* knee injuryand osteoarthritis outcome score, *HSS* Hospital for Special Surgery, *WOMAC* Western Ontarioand McMaster Universities Osteoarthritis Index, *NPRS* Numeric Pain Rating Scale,

A univariate meta-regression analysis was conducted using seven covariates—type of injury, intervention modality, intervention duration, and intervention frequency. The results indicated that none of these factors had a statistically significant effect on pain scores (*P* > 0.05), suggesting that they were not the primary sources of heterogeneity (Table 5).

**Table 5:** Meta-regression analysis results

Std_Eff	Coefficient	Std. err	t	*P*>|t|	(95% conf. interval)
Injury types	− 0.3459017	0.9828684	-0.35	0.731	-2.489567 1.797763
Intervention modality	− 0.167616	0.8133267	-0.21	0.840	-1.939703 1.604471
Intervention cycle	0.0375293	0.1352155	0.28	0.786	− 0.25708 0.3321386
Frequency of intervention	− 0.2787464	0.229565	-1.21	0.248	− 0.7789257 0.2214329

**Fig. 7:**
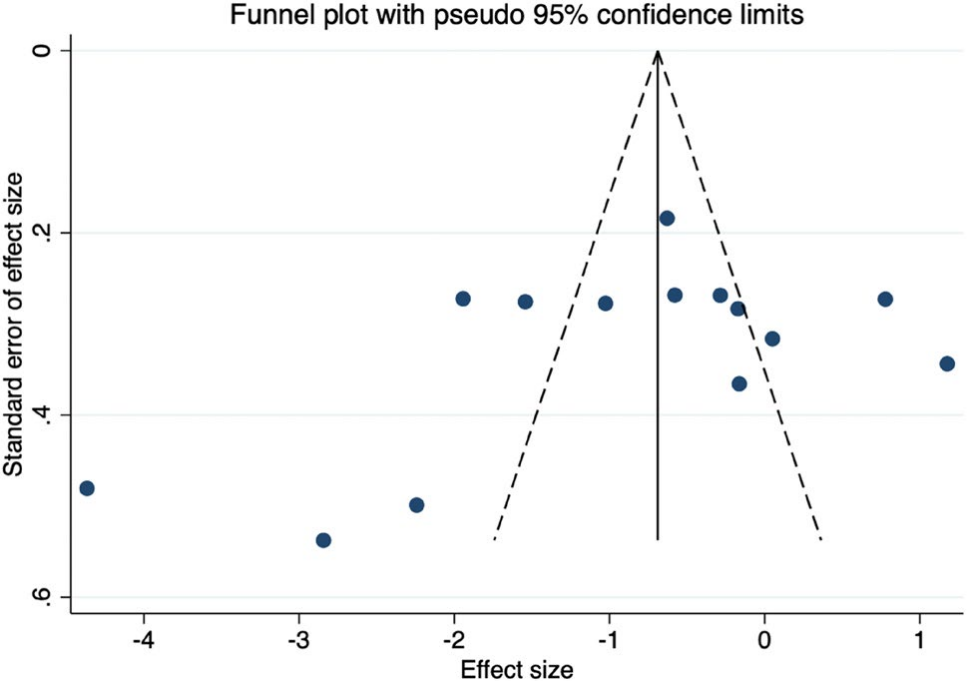
Funnel plot illustrating the effect of PNF on pain scores

Twelve studies reported the effects of PNF on pain scores, and we therefore assessed the risk of publication bias. The Egger’s test (*P* = 0.189) indicated no significant publication bias (Table 6). However, the funnel plot revealed that the data from these 12 studies did not form a symmetrical distribution, suggesting the presence of potential publication bias (Fig. 7). This discrepancy may indicate that the statistical test failed to detect a small-study effect—often associated with publication bias—while the asymmetry in the plot points to other possible sources of bias.

**Table 6:** Egger test results

Std_Eff	Coefficient	Std. err	t	*P*>|t|	(95% conf. interval)
slope	0.7908965	1.100528	0.72	0.486	-1.606946 3.18874
bias	-5.192751	3.726157	-1.39	0.189	-2.058461 2.925847

The primary reasons for this inconsistency include the relatively low statistical power of Egger’s test when applied to a small number of studies, which may limit its ability to detect true publication bias. Additionally, funnel plot asymmetry is not specific to publication bias and may arise from other factors, such as heterogeneity across studies (e.g., differences in intervention intensity, participant characteristics, or control group risk), methodological flaws in some lower-quality studies (e.g., improper blinding leading to overestimation of effect sizes), measurement errors, random variation, or the genuine non-publication of small negative studies in one direction (i.e., true publication bias). Therefore, the results should be interpreted with caution.

**Fig. 8:**
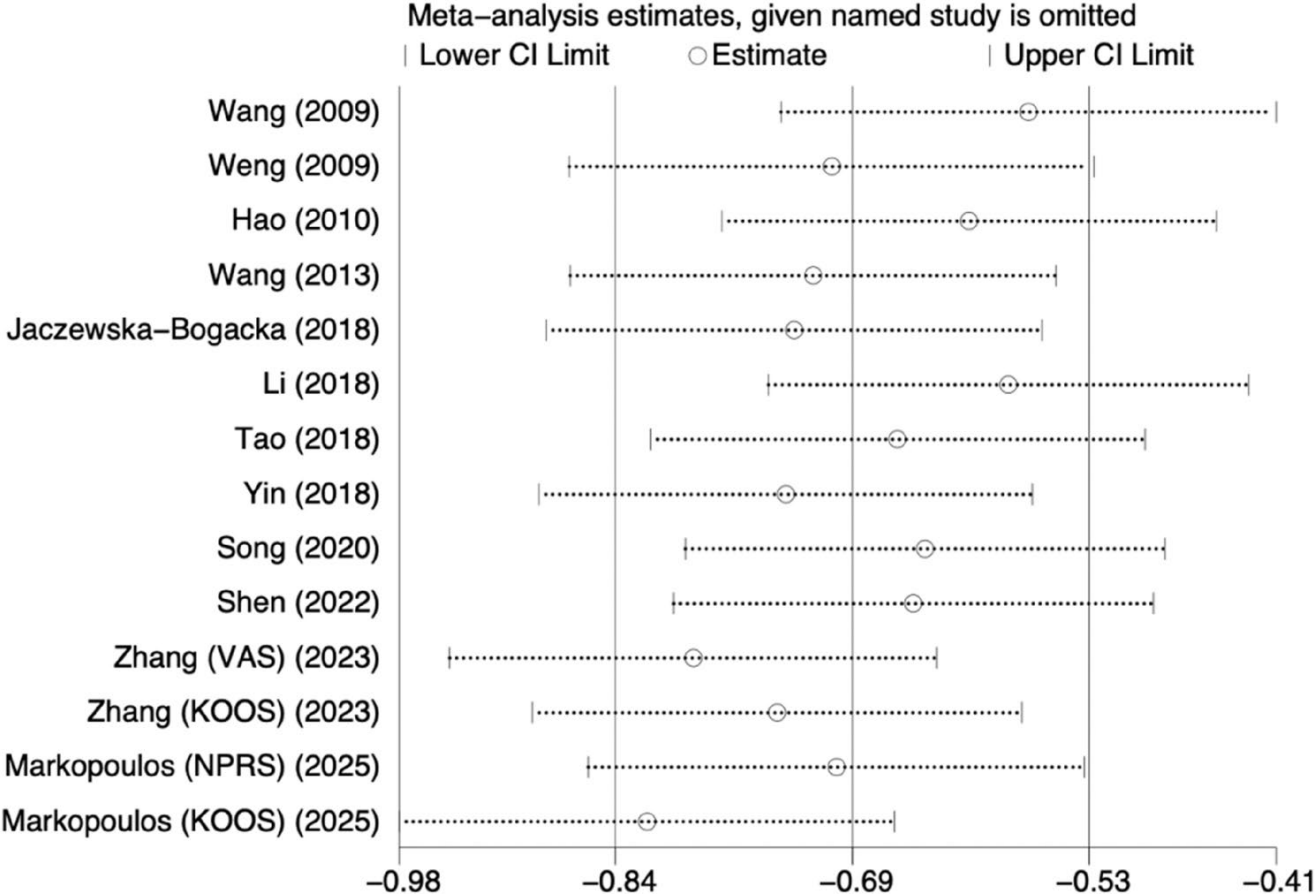
Sensitivity analysis of the effect of PNF on pain scores

A leave-one-out sensitivity analysis was performed by sequentially excluding individual studies to observe the effect on the pooled estimate. The effect sizes after excluding each study remained within the 95% confidence interval of the overall effect, indicating minimal influence on the pooled result. This suggests that the meta-analysis findings are stable and reliable (Fig. 8).

As shown in Table 4, for patients with knee joint injuries, a combined PNF intervention with a duration of 8–12 weeks (SMD = -1.31, 95% CI: -2.28 – -0.33, *P* = 0.008), performed 5–7 times per week (SMD = -1.48, 95% CI: -2.42 – -0.53, *P* = 0.002) and 10–30 min per session (SMD = -1.40, 95% CI: -2.32 – -0.49, *P* = 0.003), was more effective in alleviating pain in patients during the post-injury or postoperative recovery period (SMD = -1.23, 95% CI: -2.18 – -0.28, *P* = 0.01).

#### Effect of PNF on LKSS scores

**Fig. 9:**
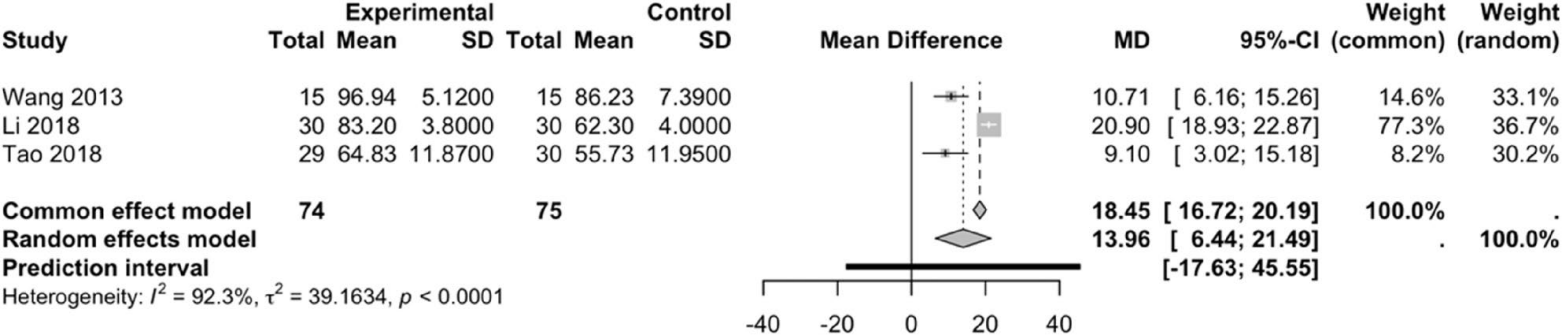
Forest plot illustrating the effect of PNF on LKSS scores

Three studies [35, 37, 39] included in this meta-analysis investigated the effect of PNF on LKSS scores. A comparison between the experimental and control groups revealed substantial heterogeneity among the included studies (*I²* = 92.3%, *P* < 0.0001); therefore, a random-effects model was applied. PNF can significantly enhance knee joint function in patients with knee injuries compared with the control group (WMD = 13.96, 95% CI: 6.44–21.49, 95%PI: -17.63–45.55) (Fig. 9).

#### Effect of PNF on BBS scores

**Fig. 10:**
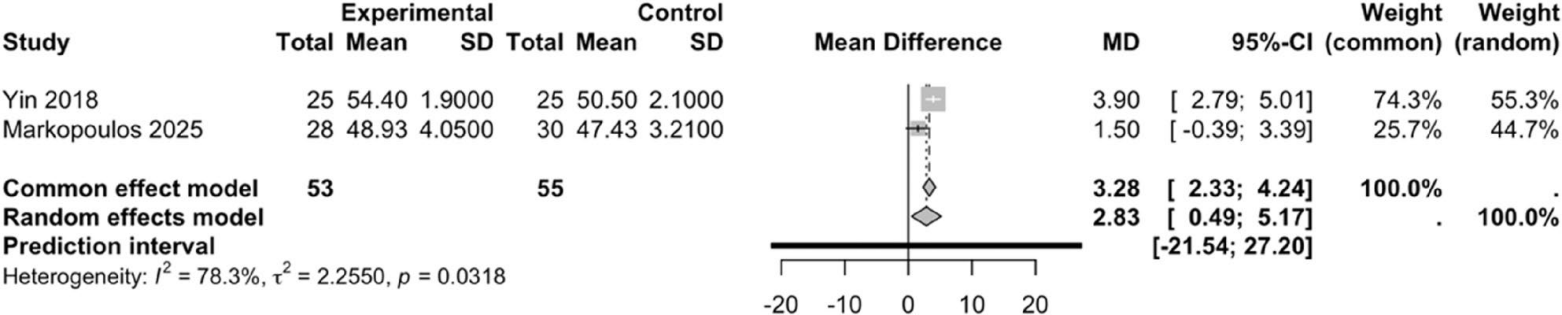
Forest plot illustrating the effect of PNF on BBS scores

Two studies [36, 43] included in this meta-analysis investigated the effect of PNF on BBS scores. A high degree of heterogeneity was observed among the study results (*I²* = 78.3%, *P* = 0.0318); therefore, a random-effects model was applied. PNF significantly improved balance in patients with knee injuries compared to the control group (WMD = 2.83, 95% CI: 0.49–5.17, 95%PI: -21.54–27.20) (Fig. 10).

#### Effect of PNF on ADL scores

**Fig. 11:**
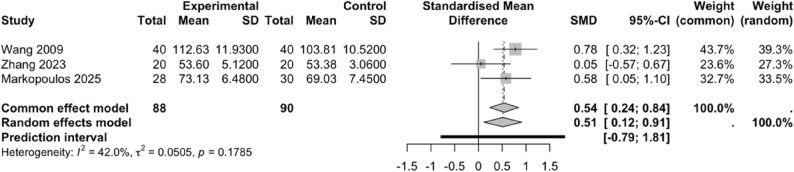
Forest plot illustrating the effect of PNF on ADL scores

Three studies [33, 42, 43] included in this meta-analysis evaluated the effect of PNF on ADL scores. A comparison between the experimental and control groups indicated mild heterogeneity among the included studies (*I²* = 42.0%, *P* = 0.1785); therefore, a fixed-effects model was applied. Among these studies, two used the KOOS scale [42, 43] and one employed the FIM scale [33], and thus the SMD was used for analysis. PNF significantly improves activities of daily living in patients with knee injuries compared to the control group (SMD = 0.51, 95% CI: 0.12–0.91, 95%PI: -0.79–1.81) (Fig. 11).

### Certainty of evidence

The certainty of evidence for one outcome measure (pain score) was rated as low, while the certainty of evidence for the remaining outcomes was rated as very low. The downgrading was primarily attributed to the risk of bias and imprecision in the included studies (Supplementary Table 3).

## Discussion

The results of this study indicate that, compared with the control group, PNF techniques significantly improve FR in patients with knee joint injuries, with a mean increase of 11.15°. This improvement is attributed to the use of spiral-diagonal movement patterns and resistance training, which activate knee proprioceptors and promote synergistic muscle coordination [44, 45]. Subgroup analysis suggests that a combined PNF intervention lasting 8–12 weeks, administered six times per week for 10–30 min per session, may be particularly effective during post-traumatic or postoperative rehabilitation. Such a regimen aligns with the “distributed practice” principle in motor learning, facilitating neuromuscular adaptation while minimizing fatigue [46, 47]. Although functional improvements have been reported after shorter interventions (e.g., 6 weeks), longer durations are associated with more sustained outcomes, as supported by RCTs demonstrating significant gains in pain, gait, and function after 8 weeks of PNF training [16, 48, 49]. High-frequency, shorter-duration sessions may thus balance efficacy, adherence, and safety [22, 43]. ER, PNF has similarly demonstrated significant rehabilitative benefits. Our study showed that patients undergoing PNF training experienced an average ER increase of 5.42°, markedly outperforming the control group. This improvement is primarily attributed to the alternating isometric contraction–relaxation patterns characteristic of PNF techniques, which effectively enhance knee extension capacity and joint stability by regulating the balance of antagonist muscle groups [50]. Notably, improvements in ER are clinically important for preventing postoperative joint stiffness and secondary osteoarthritis [51], further highlighting the unique role of PNF in comprehensive knee function recovery.

This study demonstrated that PNF significantly reduced pain in patients with knee joint injuries compared to controls. This finding is consistent with previous RCTs which employed different assessment scales, such as the VAS and WOMAC, to confirm PNF’s analgesic effect [16, 40]. The underlying mechanism may involve PNF’s ability to optimize lower-limb biomechanics, thereby reducing medial knee joint loading [40]. Furthermore, subgroup analysis suggested that a combined PNF intervention, delivered 5–7 times per week for 10–30 min per session over 8–12 weeks, appears to be a particularly effective regimen for pain relief during post-trauma or postoperative recovery, a conclusion supported by existing systematic reviews [52].

The analgesic effect of PNF is primarily mediated by its strong stimulation of muscle spindles and GTOs during contraction-relaxation, which generates non-nociceptive afferent input to inhibit spinal nociceptive transmission, providing immediate pain relief [16]. For sustained effects, a regimen of 5–7 sessions per week, each lasting 10–30 min over 8–12 weeks, aligns with the “frequency-dependent” principles of motor learning and neural plasticity, facilitating long-term proprioceptive improvement and functional recovery [52–54]. This specific prescription, while requiring further validation and individualization in clinical practice, theoretically optimizes outcomes by combining sufficient dose and duration to promote neuromuscular adaptation and may be most effective when PNF is integrated with other interventions such as strength training [15, 43].

However, it is crucial to interpret these findings as hypothesis-generating rather than conclusive. The identified parameters (e.g., 8–12 weeks, 5–7 sessions/week) represent a pattern within the available data, but they should not be construed as a definitive or optimal prescription. The high heterogeneity and limited number of studies comparing different dosages directly mean that these exploratory findings require rigorous validation in future trials specifically designed to establish dose-response relationships.

The analgesic effects of PNF may involve multiple neurophysiological mechanisms. First, PNF may activate GTOs and muscle spindles, triggering autogenic and reciprocal inhibition, thereby reducing muscle tension and attenuating pain signaling [55]. Second, contract-relax techniques in PNF may improve the viscoelastic properties of muscles and tendons via stress-relaxation mechanisms, alleviating discomfort [55]. In addition, the gate control theory may play a role, as PNF can stimulate large-diameter afferent fibers (e.g., tactile and pressure receptors) to inhibit small-diameter nociceptive fibers, thereby reducing pain perception [56]. PNF may enhance proprioceptive feedback by stimulating muscle spindles and GTOs, thereby improving sensory-motor integration and joint position sense. It can acutely reduce muscle stiffness (e.g., shear modulus), which supports a mechanical basis for increased joint range of motion [57]. At the same time, PNF likely increases stretch tolerance through modulation of nociceptive afferents, contributing to perceived pain relief [58]. Its analgesic effect is also mediated by activation of endogenous descending inhibitory systems, restoring or augmenting conditioned pain modulation [59]. Over repeated training, PNF may drive neuroplastic changes in central motor networks, optimizing coordination and neuromuscular control [60]. Finally, by improving neuromuscular control and redistributing muscular load across joints, PNF could decrease pathological stress concentrations, thus indirectly alleviating pain and promoting function [22].

For patients in the postoperative or injury recovery phase, primary goals include restoring joint range of motion, alleviating pain, and enhancing proprioception and dynamic stability. PNF, as a comprehensive approach integrating stretching, strengthening, and coordination training, can simultaneously target these objectives, thereby providing a rational basis for its use as a combined intervention in this population [46, 61]. Moreover, combining PNF with isokinetic strength training, joint mobilization, or neuromuscular training often yields superior outcomes compared to monotherapy, supporting the consideration of multi-modal strategies under the framework of “PNF in combination with other interventions” [62]. PNF stretching combined with structured exercises led to greater improvements in function, elbow flexion range of motion, and pain compared to static stretching in patients with post-traumatic elbow stiffness [63]. Similarly, integrating PNF with tuina therapy provided superior pain reduction and functional recovery in individuals with nonspecific chronic neck pain [64]. Furthermore, combining PNF with strength training resulted in significantly better outcomes in disability, pain, and grip strength than strength training alone in postmenopausal women with thumb osteoarthritis [62].

In the present study, the PNF group demonstrated significant functional improvements, as evidenced by increases in the LKSS scores (13.88 points), BBS scores (2.83 points), and ADL scores (0.54 points). These results indicate that PNF not only alleviates pain and enhances knee joint stability but also effectively improves balance and functional capacity for daily tasks. The improvement in knee function is supported by the underlying mechanisms of PNF, which can increase joint range of motion and reduce muscle tension through autogenic and reciprocal inhibition [55]. Furthermore, the observed enhancement in balance is consistent with findings that PNF training improves proprioceptive input and neuromuscular coordination [65], while the gains in daily living activities align with the principle that enhanced physical capacity facilitates superior functional performance [66].

The systematic review by Wang et al. indicated that proprioceptive training (including PNF) offers clear benefits for pain and functional improvement in patients with knee osteoarthritis, supporting its inclusion in structured rehabilitation programs [52]. Regarding patient adherence, the study by Yin et al. showed that PNF, due to its diverse operational modes and integration of functional activities, helps enhance patient participation and treatment persistence [22]. Furthermore, Campos-Villegas et al. found that combining PNF with strength training not only optimizes therapeutic efficacy, but its group-based implementation format is also easier to disseminate clinically, suggesting that the mode of intervention delivery significantly impacts treatment accessibility and effectiveness [62]. In summary, current evidence supports PNF as an effective component of knee injury rehabilitation, particularly suitable for clinical settings focusing on functional recovery and adherence management.

Although there is considerable variation in PNF intervention parameters (such as intervention frequency, session duration, and intervention period) across the included studies, this may stem from differences in the target populations, injury stages, and rehabilitation priorities of each study. However, this study identified through subgroup analysis that among patients during the trauma or postoperative recovery phase, a combined PNF intervention protocol—administered 5–7 times per week, with each session lasting 10–30 min, over a period of 8–12 weeks—demonstrated more significant effects in improving knee joint range of motion and alleviating pain. These findings suggest that PNF interventions with higher frequency, moderate session duration, and longer overall duration may better align with the principles of neuromuscular adaptation and motor learning, thereby contributing to optimized rehabilitation outcomes.

Although subgroup analysis and meta-regression were conducted to explore potential sources of heterogeneity (such as injury type, intervention modality, duration, and frequency), the results still indicated high heterogeneity, suggesting that these variables could not fully explain the variation. Potential sources of heterogeneity that may not have been adequately captured include differences in the specific technical details and operational standards of PNF, diversity in patients’ clinical characteristics and disease course, inconsistencies in control group interventions, variations in pain assessment tools and measurement time points, and limitations in the methodological quality of the studies (such as the lack of blinding and allocation concealment). In addition, differences in regional culture and rehabilitation backgrounds may also affect the consistency of the results.

While the present study did not include long-term follow-up data, the currently reviewed literature primarily focused on immediate or short-term outcomes following the intervention. Therefore, future research should be designed with randomized controlled trials that include long-term follow-up periods (e.g., 6 months, 12 months post-intervention) to assess the sustained benefits and potential delayed effects of PNF on knee range of motion, pain, function, and activities of daily living. Additionally, investigating the long-term dose-response relationship of different PNF protocols (such as intervention duration, frequency, and session length), as well as the role of PNF in preventing re-injury and slowing the progression of osteoarthritis, will be valuable directions for further research.

## Limitations

(1) High heterogeneity (I² > 90%) remains a major limitation, likely due to varying PNF techniques and control treatments. (2) Differences in intervention protocols, outcome measures, and small sample sizes further contribute to variability. Thus, subgroup findings should be viewed as evidence-informed suggestions rather than definitive guidelines. (3) Methodological reporting was generally poor, with inadequate description of randomization, blinding, and allocation concealment, leading to an overall high risk of bias. (4) Geographic bias is a concern, as most studies were from China (10/13), limiting generalizability. (5) The use of multiple pain scales may introduce measurement bias. (6) Funnel plot asymmetry indicates potential publication bias despite a non-significant Egger’s test. (7) The sources of heterogeneity for pain outcomes could not be conclusively identified.

## Conclusion

PNF can significantly improve the active flexion and extension range of motion, reduce pain, and enhance knee function, balance, and activities of daily living in patients with knee injuries. Based on exploratory subgroup findings, an intervention lasting 8–12 weeks, performed 5–7 times per week for 10–30 min per session, combining PNF with other therapies, may be more effective in alleviating pain and improving knee flexion range in patients with knee injuries or during postoperative recovery. However, Given the limited certainty of the existing certainty of evidence, the interpretation of these findings necessitates caution, confirming its efficacy and establishing definitive dosage guidelines require validation in future trials specifically designed to compare different PNF dosages.

## Supplementary Information

Supplementary Material 1.

## Data Availability

The datasets used and analyzed during the current study are available from the corresponding author on reasonable request.
