# Supplementary material for: The effect of Proprioceptive Neuromuscular Facilitation (PNF) therapy on functional recovery in patients with knee joint injury: a systematic review and meta-analysis
Source: BMC Musculoskelet Disord. 2025 Dec 17;27:47. doi: 10.1186/s12891-025-09407-z (PMC12821984; doi:10.1186/s12891-025-09407-z)
Supplement: Supplementary file 1 — Supplementary Material 1. [file 12891_2025_9407_MOESM1_ESM.docx]

# **Supplementary Tables**

**Supplementary Table S1.** The Preferred Reporting Items for Systematic reviews and Meta-Analysis (PRISMA) 2020 Main Checklist.

| **Topic** | **No.** | **Item** | **Location where item is reported** |
| --- | --- | --- | --- |
| **TITLE** |  |  |  |
| **Title** | 1 | Identify the report as a systematic review. | Title |
| **ABSTRACT** |  |  |  |
| **Abstract** | 2 | See the PRISMA 2020 for Abstracts checklist | Abstract |
| **INTRODUCTION** |  |  |  |
| **Rationale** | 3 | Describe the rationale for the review in the context of existing knowledge. | 1 Introduction |
| **Objectives** | 4 | Provide an explicit statement of the objective(s) or question(s) the review addresses. | 1 Introduction |
| **METHODS** |  |  |  |
| **Eligibility criteria** | 5 | Specify the inclusion and exclusion criteria for the review and how studies were grouped for the syntheses. | 2.3 Inclusion and exclusion criteria for the studies |
| **Information sources** | 6 | Specify all databases, registers, websites, organizations, reference lists and other sources searched or consulted to identify studies. Specify the date when each source was last searched or consulted. | 2.2 Search strategy |
| **Search strategy** | 7 | Present the full search strategies for all databases, registers and websites, including any filters and limits used. | 2.2 Search strategy |
| **Selection process** | 8 | Specify the methods used to decide whether a study met the inclusion criteria of the review, including how many reviewers screened each record and each report retrieved, whether they worked independently, and if applicable, details of automation tools used in the process. | 2.4 Literature screening and data extraction |
| **Data collection process** | 9 | Specify the methods used to collect data from reports, including how many reviewers collected data from each report, whether they worked independently, any processes for obtaining or confirming data from study investigators, and if applicable, details of automation tools used in the process. | 2.4 Literature screening and data extraction |
| **Data items** | 10a | List and define all outcomes for which data were sought. Specify whether all results that were compatible with each outcome domain in each study were sought (e.g. for all measures, time points, analyses), and if not, the methods used to decide which results to collect. | 2.3 Inclusion and exclusion criteria for the studies  2.4 Literature screening and data extraction |
|  | 10b | List and define all other variables for which data were sought (e.g. participant and intervention characteristics, funding sources). Describe any assumptions made about any missing or unclear information. | 2.4 Literature screening and data extraction |
| **Study risk of bias assessment** | 11 | Specify the methods used to assess risk of bias in the included studies, including details of the tool(s) used, how many reviewers assessed each study and whether they worked independently, and if applicable, details of automation tools used in the process. | 2.5 Risk of bias assessment |
| **Effect measures** | 12 | Specify for each outcome the effect measure(s) (e.g. risk ratio, mean difference) used in the synthesis or presentation of results. | 2.6 Data analysis |
| **Synthesis methods** | 13a | Describe the processes used to decide which studies were eligible for each synthesis (e.g. tabulating the study intervention characteristics and comparing against the planned groups for each synthesis (item 5)). | Table 1. Research characteristics  Table 2. Characterization of research interventions |
|  | 13b | Describe any methods required to prepare the data for presentation or synthesis, such as handling of missing summary statistics, or data conversions. | 2.6 Data analysis |
|  | 13c | Describe any methods used to tabulate or visually display results of individual studies and syntheses. | Table 1. Research characteristics  Table 2. Characterization of research interventions |
|  | 13d | Describe any methods used to synthesize results and provide a rationale for the choice(s). If meta-analysis was performed, describe the model(s), method(s) to identify the presence and extent of statistical heterogeneity, and software package(s) used. | 2.6 Data analysis |
|  | 13e | Describe any methods used to explore possible causes of heterogeneity among study results (e.g. subgroup analysis, meta-regression). | 2.7 Subgroup analysis |
|  | 13f | Describe any sensitivity analyses conducted to assess robustness of the synthesized results. | 2.8 Sensitivity analysis |
| **Reporting bias assessment** | 14 | Describe any methods used to assess risk of bias due to missing results in a synthesis (arising from reporting biases). | 2.5 Risk of bias assessment |
| **Certainty assessment** | 15 | Describe any methods used to assess certainty (or confidence) in the body of evidence for an outcome. | 2.11 Certainty of evidence |
| **RESULTS** |  |  |  |
| **Study selection** | 16a | Describe the results of the search and selection process, from the number of records identified in the search to the number of studies included in the review, ideally using a flow diagram. | 3.1 Results of literature screening  Figure1.  PRISMA study flow diagram. |
|  | 16b | Cite studies that might appear to meet the inclusion criteria, but which were excluded, and explain why they were excluded. | 3.1 Results of literature screening |
| **Study characteristics** | 17 | Cite each included study and present its characteristics. | 3.2 Characteristics of the included studies |
| **Risk of bias in studies** | 18 | Present assessments of risk of bias for each included study. | 3.3 Risk of bias |
| **Results of individual studies** | 19 | For all outcomes, present, for each study: (a) summary statistics for each group (where appropriate) and (b) an effect estimates and its precision (e.g. confidence/credible interval), ideally using structured tables or plots. | 3.4 Meta-analysis results |
| **Results of syntheses** | 20a | For each synthesis, briefly summaries the characteristics and risk of bias among contributing studies. | 3.3 Risk of bias  3.4 Meta-analysis results |
|  | 20b | Present results of all statistical syntheses conducted. If meta-analysis was done, present for each the summary estimate and its precision (e.g. confidence/credible interval) and measures of statistical heterogeneity. If comparing groups, describe the direction of the effect. | 3.4 Meta-analysis results |
|  | 20c | Present results of all investigations of possible causes of heterogeneity among study results. | 3.4 Meta-analysis results |
|  | 20d | Present results of all sensitivity analyses conducted to assess the robustness of the synthesized results. | 3.4 Meta-analysis results |
| **Reporting biases** | 21 | Present assessments of risk of bias due to missing results (arising from reporting biases) for each synthesis assessed. | - |
| **Certainty of evidence** | 22 | Present assessments of certainty (or confidence) in the body of evidence for each outcome assessed. | 3.5 Certainty of evidence |
| **DISCUSSION** |  |  |  |
| **Discussion** | 23a | Provide a general interpretation of the results in the context of other evidence. | 4 Discussion |
|  | 23b | Discuss any limitations of the evidence included in the review. | 4 Discussion |
|  | 23c | Discuss any limitations of the review processes used. | 4 Discussion |
|  | 23d | Discuss implications of the results for practice, policy, and future research. | 4 Discussion |
| **OTHER INFORMATION** |  |  |  |
| **Registration and protocol** | 24a | Provide registration information for the review, including register name and registration number, or state that the review was not registered. | 2.1 Protocol and registration |
|  | 24b | Indicate where the review protocol can be accessed, or state that a protocol was not prepared. | 2.1 Protocol and registration |
|  | 24c | Describe and explain any amendments to information provided at registration or in the protocol. | 2.1 Protocol and registration |
| **Support** | 25 | Describe sources of financial or non-financial support for the review, and the role of the funders or sponsors in the review. | Funding information |
| **Competing interests** | 26 | Declare any competing interests of review authors. | Declaration of competing interest |
| **Availability of data, code and other materials** | 27 | Report which of the following are publicly available and where they can be found template data collection forms; data extracted from included studies; data used for all analyses; analytic code; any other materials used in the review. | Availability of data and materials |

**Supplementary Table S2.** Database search strategy.

| Database | Search strategies |
| --- | --- |
| China National Knowledge Infrastructure (CNKI) | SU=(本体感觉神经肌肉促进法+PNF+PNF拉伸+被动拉伸+静力-被动拉伸+放松拉伸+静态拉伸+等长拉伸+动态拉伸+弹震式拉伸+自生抑制+交互抑制+节律性稳定+重复收缩+保持-放松+拮抗肌收缩+慢逆转+功能性牵张反射+反射兴奋性+收缩-放松+Kabat) AND SU=(膝损伤+膝关节运动损伤+膝骨关节炎+前交叉韧带重建) AND SU=(随机对照试验+对照临床试验+临床试验+临床研究) |
| PubMed | ((((((((((((((((((proprioceptive neuromuscular facilitation[MeSH]) OR (PNF[Title/Abstract])) OR (proprioceptive neuromuscular facilitation (PNF) stretching[Title/Abstract])) OR (passive stretching[Title/Abstract])) OR (static-passive stretching[Title/Abstract])) OR (relaxed stretching[Title/Abstract])) OR (static stretching[Title/Abstract])) OR (isometric stretching[Title/Abstract])) OR (dynamic stretching[Title/Abstract])) OR (ballistic stretching[Title/Abstract])) OR (autogenic inhibition[Title/Abstract])) OR (reciprocal inhibition[Title/Abstract])) OR (rhythmic stabilization[Title/Abstract])) OR (repeated contraction[Title/Abstract])) OR (hold-relax[Title/Abstract])) OR (antagonist contract[Title/Abstract])) OR (slow reversal[Title/Abstract])) OR (functional stretch reflex[Title/Abstract])) OR (reflex excitability[Title/Abstract])) OR (contract-relax[Title/Abstract])) OR (Kabat[Title/Abstract])))  AND  (((((((knee injury[MeSH]) OR (knee joint sports injury[Title/Abstract])) OR (knee osteoarthritis[Title/Abstract])) OR (anterior cruciate ligament reconstruction[Title/Abstract])) OR (ACL reconstruction[Title/Abstract])) OR (knee trauma[Title/Abstract])) OR (knee surgery[Title/Abstract])))  AND  ((((randomized controlled trial[MeSH]) OR (controlled clinical trial[Title/Abstract])) OR (clinical trial[Title/Abstract])) OR (clinical study[Title/Abstract]))) |
| Embase | ('proprioceptive neuromuscular facilitation'/de OR 'PNF':ti,ab OR 'proprioceptive neuromuscular facilitation (PNF) stretching':ti,ab OR 'passive stretching':ti,ab OR 'static-passive stretching':ti,ab OR 'relaxed stretching':ti,ab OR 'static stretching':ti,ab OR 'isometric stretching':ti,ab OR 'dynamic stretching':ti,ab OR 'ballistic stretching':ti,ab OR 'autogenic inhibition':ti,ab OR 'reciprocal inhibition':ti,ab OR 'rhythmic stabilization':ti,ab OR 'repeat contraction':ti,ab OR 'hold relax':ti,ab OR 'antagonist contract':ti,ab OR 'slow reversal':ti,ab OR 'functional stretch reflex':ti,ab OR 'reflex excitability':ti,ab OR 'contract relax':ti,ab OR 'kabat':ti,ab) AND ('knee injury'/de OR 'knee joint sports injury':ti,ab OR 'knee osteoarthritis':ti,ab OR 'anterior cruciate ligament reconstruction':ti,ab) AND ('randomized controlled trial'/de OR 'controlled clinical trial':ti,ab OR 'clinical trial':ti,ab OR 'clinical study':ti,ab) |
| Cochrane library | (proprioceptive neuromuscular facilitation[MeSH] OR PNF[tiab] OR "proprioceptive neuromuscular facilitation"[tiab] OR "PNF stretching"[tiab] OR "passive stretching"[tiab] OR "static-passive stretching"[tiab] OR "relaxed stretching"[tiab] OR "static stretching"[tiab] OR "isometric stretching"[tiab] OR "dynamic stretching"[tiab] OR "ballistic stretching"[tiab] OR "autogenic inhibition"[tiab] OR "reciprocal inhibition"[tiab] OR "rhythmic stabilization"[tiab] OR "repeat contraction"[tiab] OR "hold relax"[tiab] OR "antagonist contract"[tiab] OR "slow reversal"[tiab] OR "functional stretch reflex"[tiab] OR "reflex excitability"[tiab] OR "contract relax"[tiab] OR kabat[tiab])  AND  (knee injuries[MeSH] OR "knee injury"[tiab] OR "knee injuries"[tiab] OR "knee joint sports injury"[tiab] OR "knee osteoarthritis"[tiab] OR "osteoarthritis, knee"[MeSH] OR "anterior cruciate ligament reconstruction"[tiab] OR "anterior cruciate ligament injuries"[MeSH])  AND  (randomized controlled trial[pt] OR controlled clinical trial[pt] OR randomized[tiab] OR placebo[tiab] OR "clinical trial"[tiab] OR "randomly"[tiab] OR "trial"[tiab] OR "clinical study"[tiab]) |
| Web of Science | TS=(("proprioceptive neuromuscular facilitation" OR PNF OR "proprioceptive neuromuscular facilitation stretching" OR "PNF stretching" OR "passive stretching" OR "static-passive stretching" OR "relaxed stretching" OR "static stretching" OR "isometric stretching" OR "dynamic stretching" OR "ballistic stretching" OR "autogenic inhibition" OR "reciprocal inhibition" OR "rhythmic stabilization" OR "repeat contraction" OR "hold relax" OR "antagonist contract" OR "slow reversal" OR "functional stretch reflex" OR "reflex excitability" OR "contract relax" OR kabat))  AND  TS=(("knee injury" OR "knee injuries" OR "knee joint sports injury" OR "knee osteoarthritis" OR "osteoarthritis, knee" OR "anterior cruciate ligament reconstruction" OR "ACL reconstruction"))  AND  TS=(("randomized controlled trial" OR "controlled clinical trial" OR "clinical trial" OR "clinical study" OR randomly OR trial)) |

**Supplementary Table S3.** Certainy of evidence.

**Author(s):** Wenhua Zhang

**Question:** [exercise] compared to [no PNF] for [functional recovery in patients with knee joint injury]

**Bibliography:** Zhang Y, Akl EA, Schünemann HJ. Using systematic reviews in guideline development: the GRADE approach. Res Synth Methods. 2019;10(3).

| **Certainty assessment** | | | | | | | **№ of patients** | | **Effect** | | **Certainty** | **Importance** |
| --- | --- | --- | --- | --- | --- | --- | --- | --- | --- | --- | --- | --- |
| **№ of studies** | **Study design** | **Risk of bias** | **Inconsistency** | **Indirectness** | **Imprecision** | **Other considerations** | **[PNF]** | **[no PNF]** | **Relative (95% CI)** | **Absolute (95% CI)** |  |  |
| **FR** | | | | | | | | | | | | |
| 7 | randomised trials | very serious^a,b,c^ | not serious | not serious | serious^d^ | none | 214 | 213 | - | MD **11.15 SD higher** (2.91 higher to 19.38 higher) | ⨁◯◯◯ Very low^a,b,c,d^ |  |
| **ER** | | | | | | | | | | | | |
| 2 | randomised trials | very serious^a,b,c^ | not serious | not serious | serious^d^ | none | 77 | 72 | - | MD **5.42 SD higher** (1.29 higher to 9.56 higher) | ⨁◯◯◯ Very low^a,b,c,d^ |  |
| **Pain score** | | | | | | | | | | | | |
| 12 | randomised trials | very serious^a,b,c^ | not serious | not serious | not serious | none | 389 | 391 | - | SMD **1.08 SD lower** (1.63 lower to 0.54 lower) | ⨁⨁◯◯ Low^a,b,c^ |  |
| **Lysholm knee joint function score** | | | | | | | | | | | | |
| 3 | randomised trials | very serious^a,b,c^ | not serious | not serious | serious^d^ | none | 74 | 75 | - | MD **13.88 higher** (5.44 higher to 22.32 higher) | ⨁◯◯◯ Very low^a,b,c,d^ |  |
| **BBS score** | | | | | | | | | | | | |
| 2 | randomised trials | very serious^a,b,c^ | not serious | not serious | serious^d^ | none | 53 | 55 | - | MD **2.83 higher** (0.49 higher to 5.17 higher) | ⨁◯◯◯ Very low^a,b,c,d^ |  |
| **ADL score** | | | | | | | | | | | | |
| 3 | randomised trials | very serious^a,b,c^ | not serious | not serious | serious^d^ | none | 88 | 90 | - | SMD **0.54 SD higher** (0.24 higher to 0.84 higher) | ⨁◯◯◯ Very low^a,b,c,d^ |  |

#### **CI**: confidence interval; **MD**: mean difference; **SMD**: standardised mean difference; **FR**: flexion range; **ER**: extension range; **BBS**: berg balance scale; **LKSS**: Lysholm knee score scale; **ADL**: activity of daily living

#### Explanations

a. Some studies did not provide specific randomization methods

b. Don't blind researchers and participants in experiments or provided insufficient information

c. Provide insufficient information on blinding of outcome assessment or high risk for blinding of outcome assessment

d. The sample size is low
